# Unmasking Pediatric Asthma: Epigenetic Fingerprints and Markers of Respiratory Infections

**DOI:** 10.3390/ijms26157629

**Published:** 2025-08-06

**Authors:** Alessandra Pandolfo, Rosalia Paola Gagliardo, Valentina Lazzara, Andrea Perri, Velia Malizia, Giuliana Ferrante, Amelia Licari, Stefania La Grutta, Giusy Daniela Albano

**Affiliations:** 1 Department of Economics, Business and Statistics (dSEAS), University of Palermo (UNIPA), 90128 Palermo, Italy; alessandra.pandolfo@ift.cnr.it (A.P.); valentina.lazzara@ift.cnr.it (V.L.); 2 Institute of Translational Pharmacology (IFT), National Research Council (CNR), 90146 Palermo, Italy; rosaliapaola.gagliardo@ift.cnr.it (R.P.G.); andrea.perri@ift.cnr.it (A.P.); velia.malizia@ift.cnr.it (V.M.);; 3 Department of Earth and Marine Sciences (DiSTeM), University of Palermo (UNIPA), 90123 Palermo, Italy; 4 Department of Surgery, Dentistry, Pediatrics and Gynaecology, Pediatric Division, University of Verona, 37134 Verona, Italy; 5 Pediatric Unit, Department of Clinical, Surgical, Diagnostic and Pediatric Sciences, University of Pavia, 27100 Pavia, Italy; 6 Pediatric Clinic, Fondazione IRCCS Policlinico, San Matteo, 27100 Pavia, Italy

**Keywords:** pediatric asthma, epigenetics, asthma endotypes, respiratory infections, personalized medicine

## Abstract

Pediatric asthma is a multifactorial and heterogeneous disease determined by the dynamic interplay of genetic susceptibility, environmental exposures, and immune dysregulation. Recent advances have highlighted the pivotal role of epigenetic mechanisms, in particular, DNA methylation, histone modifications, and non-coding RNAs, in the regulation of inflammatory pathways contributing to asthma phenotypes and endotypes. This review examines the role of respiratory viruses such as respiratory syncytial virus (RSV), rhinovirus (RV), and other bacterial and fungal infections that are mediators of infection-induced epithelial inflammation that drive epithelial homeostatic imbalance and induce persistent epigenetic alterations. These alterations lead to immune dysregulation, remodeling of the airways, and resistance to corticosteroids. A focused analysis of T2-high and T2-low asthma endotypes highlights unique epigenetic landscapes directing cytokines and cellular recruitment and thereby supports phenotype-specific aspects of disease pathogenesis. Additionally, this review also considers the role of miRNAs in the control of post-transcriptional networks that are pivotal in asthma exacerbation and the severity of the disease. We discuss novel and emerging epigenetic therapies, such as DNA methyltransferase inhibitors, histone deacetylase inhibitors, miRNA-based treatments, and immunomodulatory probiotics, that are in preclinical or early clinical development and may support precision medicine in asthma. Collectively, the current findings highlight the translational relevance of including pathogen-related biomarkers and epigenomic data for stratifying pediatric asthma patients and for the personalization of therapeutic regimens. Epigenetic dysregulation has emerged as a novel and potentially transformative approach for mitigating chronic inflammation and long-term morbidity in children with asthma.

## 1. Introduction

Asthma is a heterogeneous lung disease characterized by coughing, breathlessness, wheezing, and chest tightness, resulting from chronic airway inflammation, airflow obstruction, and bronchial hyperresponsiveness. Globally, it affects approximately 9.1% of children and 11.0% of adolescents, with a mortality rate of 0.51 per 100,000 [1]. In the pediatric population, it remains a leading cause of hospitalizations and school absenteeism [2], which constitutes a major cost to healthcare systems and fosters socioeconomic costs that should not be underrated [3]. Asthma can further be defined into a broad spectrum of phenotypes and endotypes [4], although the exact causes and pathogenesis of the disease are complex and multifactorial, including genetic, environmental, and immunological elements. Asthma is conventionally categorized into two broad phenotypes according to patterns of inflammation, namely, T2-high and T2-low asthma. However, in children, these categories often overlap, and many patients present with mixed or shifting inflammatory profiles depending on age, atopic status, and environmental exposures [5].

T2-high asthma is characterized by elevated eosinophil levels in peripheral blood and sputum; activation of Th-2 lymphocytes and innate lymphocytes (ILCs); and the release of proinflammatory cytokines such as interleukin (IL)-4, IL-5, and IL-13. This phenotype typically responds well to inhaled corticosteroids and biologic therapies and is associated with epithelial thickening, mucus hypersecretion, smooth muscle hypercontractility, and increased IgE production [6,7]. In contrast, T2-low asthma, often driven by neutrophils, is generally less responsive to standard treatments, making it more challenging to manage [8].

This distinction underscores the need for personalized asthma treatment strategies that specifically target the underlying inflammatory mechanisms [7]. Interestingly, in pediatric populations, different asthma profiles have been observed, including (a) type-2 (Th-2) high and (b) type 2 (Th-2) low, which exhibit variable infection rates and differing degrees of responsiveness to inhaled corticosteroids (ICS) [9,10,11].

In addition to this phenotypic diversity, epigenetic modifications, including DNA methylation, histone modifications, and non-coding RNAs, appear to play a central role in the development of asthma by regulating key genes involved in immune response, airway remodeling, and chronic inflammation [12]. Environmental exposures, such as allergens, respiratory infections, pollutants, and dietary components, interact with epigenetic mechanisms to modify asthma susceptibility and the risk of exacerbation [13,14,15]. Understanding these epigenetic processes is essential for the development of novel therapeutic strategies. The identification of specific epigenetic markers associated with asthma phenotypes and endotypes may enable the implementation of personalized medicine approaches, with treatments tailored to individual epigenetic profiles.

Furthermore, elucidating how pathogens such as respiratory syncytial virus (RSV) and rhinovirus (RV), along with environmental exposures, influence the epigenome could lead to new insights into prevention strategies and early intervention.

In this review, we discuss the complex relationship between epigenetic mechanisms, respiratory pathogens, and pediatric asthma, showing how these interactions drive disease pathogenesis and offer novel therapeutic opportunities. We investigate the molecular changes responsible for respiratory infections in early life that alter immune responses and contribute to the initiation and progression of asthma. We also discuss the characterization of asthma endotypes according to epigenetic signature and the implications for personalized treatments. Lastly, we emphasize new therapeutic strategies directed at epigenetic modifications, which provide potential opportunities for better and personalized treatment in children with asthma.

## 2. Asthma Endotypes

Endotypes, characterized by specific pathophysiological processes, have recently been identified as a central driver to push personalized medicine forward among the numerous asthma subtypes [16]. This idea goes beyond clinical symptoms and allows for the detection of molecule-based signatures to provide personalized medication. In this regard, epigenetic modifications have been crucial in identifying endotype-specific pathways, providing new insights into disease mechanisms and enabling the development of targeted treatments.

Airway epithelial cells display distinct differentiation and epigenetic programs across asthma endotypes. In T2-high asthma, goblet cell metaplasia and epigenetic activation of alarmin (IL-25, IL-33, and TSLP) and Th2-related loci are prominent. In contrast, non–T2 asthma shows basal cell hyperplasia, barrier impairment, and chromatin/methylation changes that favor neutrophil chemoattractant genes (e.g., CXCL8). These epithelial trajectories underscore the epithelium’s role in driving endotype heterogeneity [17].

### 2.1. T2-High Asthma Endotype

The T2-high immune response is more common in pediatric populations and represents the most well-characterized asthma endotype, which is frequently associated with atopy and allergic sensitization. It is primarily driven by epithelial-derived alarmins, including IL-33, IL-25, and thymic stromal lymphopoietin (TSLP), which are released in response to allergens and respiratory pathogens [18,19]. These cytokines act on type 2 innate lymphoid cells (ILC2s), promoting the secretion of IL-5 and IL-13, which drive eosinophilic inflammation and airway hyperresponsiveness [20]. Among these, TSLP plays a key role by activating dendritic cells that promote Th2 differentiation and the production of IL-4, IL-5, IL-9, and IL-13 [21,22,23,24]. IL-33 directly stimulates both ILC2s and Th2 cells, amplifying type 2 inflammation, while IL-25 enhances ILC2 expansion and eosinophilic recruitment [25,26]. The interplay between these alarmins and immune cells sustains chronic airway inflammation, mucus hypersecretion, and structural remodeling of the airway.

Mast cells and B cells also contribute critically to T2-high asthma pathogenesis. IL-4 induces IgE class switching in B cells, leading to IgE-mediated mast cell activation. Upon degranulation, mast cells release histamine and lipid mediators, intensifying bronchoconstriction and inflammation [27,28]. These mechanisms underlie the classic eosinophilic asthma phenotypes, which generally respond well to corticosteroids and biologics targeting type 2 inflammation [29]. However, some patients may exhibit mixed eosinophilic–neutrophilic inflammation, which is characterized by persistent airway involvement and greater disease severity [30], often associated with corticosteroid resistance [31]. Mixed phenotypes, which exhibit features of both eosinophilic and neutrophilic inflammation, are increasingly recognized in clinical practice. These forms are often associated with more severe disease and corticosteroid resistance, highlighting their relevance for prognosis and treatment planning.

Epigenetic research has provided key insights into the molecular regulation of this endotype. DNA methylation changes have been shown to influence the expression of genes involved in the T2-inflammatory cascade. For example, aberrant methylation patterns, including hypermethylation and hypomethylation of genes such as GATA-3 and IL-4, have been implicated in the dysregulation of type 2 immune responses [32,33]. Epigenetic modifications in the nasal epithelium have emerged as promising biomarkers, with several studies identifying multiple methylated CpG islands in promoters of Th2-related genes [34]. Comparable findings have been reported in buccal samples, supporting the utility of accessible tissues for epigenetic profiling [35,36]. However, despite their potential, these biomarkers are not yet validated for clinical use and remain confined to research settings, pending further standardization and large-scale validation across diverse populations.

These alterations can either enhance or repress inflammatory gene expression, contributing to the chronic inflammation state characteristic of this endotype [37]. Biomarkers such as IgE levels and fractional exhaled nitric oxide (FeNO) have been associated with increased nasal epithelial methylation changes [34]. Moreover, accelerated epigenetic aging in nasal samples has been correlated with asthma and elevated markers of allergic disease [34]. The identification of these epigenetic signatures, whether in the nasal epithelium or in Th-2-associated immune cells, offers a valuable opportunity for stratifying patients, monitoring disease progression, and guiding therapeutic interventions targeting the Th2 endotype in pediatric asthma [12,38].

### 2.2. T2-Low Asthma Endotype

T2-low asthma, also known as non-T2 asthma, represents a distinct inflammatory endotype characterized by neutrophilic or paucigranulocytic airway inflammation [39,40]. Unlike the eosinophilic and corticosteroid-responsive T2-high endotype [41], T2-low asthma is often steroid-resistant and associated with alternative immunological and epigenetic mechanisms. This endotype is frequently linked to more severe, treatment-refractory disease phenotypes, underscoring the urgent need for novel therapeutic strategies [42]. In contrast to T2-high asthma, epithelial-derived alarmins such as IL-33, IL-25, and TSLP play a less prominent role in T2-low asthma. Instead, antigen-presenting cells (APCs) activate Th1 and Th17 lymphocytes, promoting a proinflammatory milieu characterized by neutrophilic infiltration [43]. Th1 cells release interferon-gamma (IFN-γ), while Th17 cells secrete interleukin-17 (IL-17), both of which contribute to persistent airway inflammation and structural remodeling [44,45]. Clinically, T2-low asthma is marked by mucus hypersecretion, epithelial barrier dysfunction, and sustained neutrophilic inflammation, features that contribute to its chronic and refractory nature [46]. At the molecular level, NF-κB and STAT3 signaling pathways are critically involved in maintaining inflammation and driving tissue remodeling in the airway epithelium [47,48,49]. Their activation contributes to corticosteroid insensitivity and exacerbates epithelial damage.

Consequently, research efforts have been targeted to neutrophil-driven inflammation and upstream regulators that result in more effective therapeutic options [50]. Epigenetic studies further support the distinctiveness of the T2-low endotype [40]. Genome-wide DNA methylation profiling studies have shown differentially methylated genes related to Th1 and Th17 responses as IFN-γ and IL-17A, further supporting the importance of these genes in the pathogenesis of T2-low asthma [51,52]. Moreover, epigenetic alterations in genes involved in neutrophil recruitment, such as CXCL8/IL-8 and CXCL1, have been directly linked to the sustained neutrophilic inflammation observed in this asthma subtype [53]. These findings, primarily derived from pediatric studies, suggest that epigenetic dysregulation plays a significant role in the steroid-resistant nature of T2-low asthma, reinforcing the need for novel therapeutic strategies that target these molecular mechanisms [37,42,53,54]. It is important to note that despite growing evidence, current pediatric-specific data on T2-low asthma endotypes remain limited, particularly concerning longitudinal outcomes and therapeutic responsiveness. With further understanding of immunological and epigenetic mechanisms driving T2-low asthma, precision medicine approaches are increasingly emerging. Unlike T2-high asthma, this endotype currently lacks approved biologic therapies specifically targeting its underlying mechanisms.

Therapies targeting IL-17, such as secukinumab and brodalumab, were initially considered promising due to their potential to modulate neutrophilic inflammation. However, clinical trials yielded disappointing results, since no significant improvements in lung function or symptom control were reported. Furthermore, safety concerns, including psychiatric adverse events, have restrained further development [55,56]. Also, IL-6-targeted therapies have emerged as potential candidates, particularly for patients with metabolic dysfunction or obesity-associated asthma. Elevated IL-6 levels correlate with steroid resistance and enhanced Th17 responses. Although agents such as tocilizumab and clazakizumab demonstrated preliminary efficacy in certain cases, robust clinical data remain limited [56,57]. The AMAZES trial has confirmed the benefits of neutrophil-directed therapies, such as long-term macrolide (azithromycin) administration, in reducing exacerbation rates in patients with neutrophilic inflammation. However, these therapies should be used with caution due to antimicrobial resistance concerns [58].

Other novel approaches include APOA1 mimetic peptides and type I interferons, though these remain in preclinical or early clinical phases, and their therapeutic applicability is yet to be validated [11].

**Figure 1:**
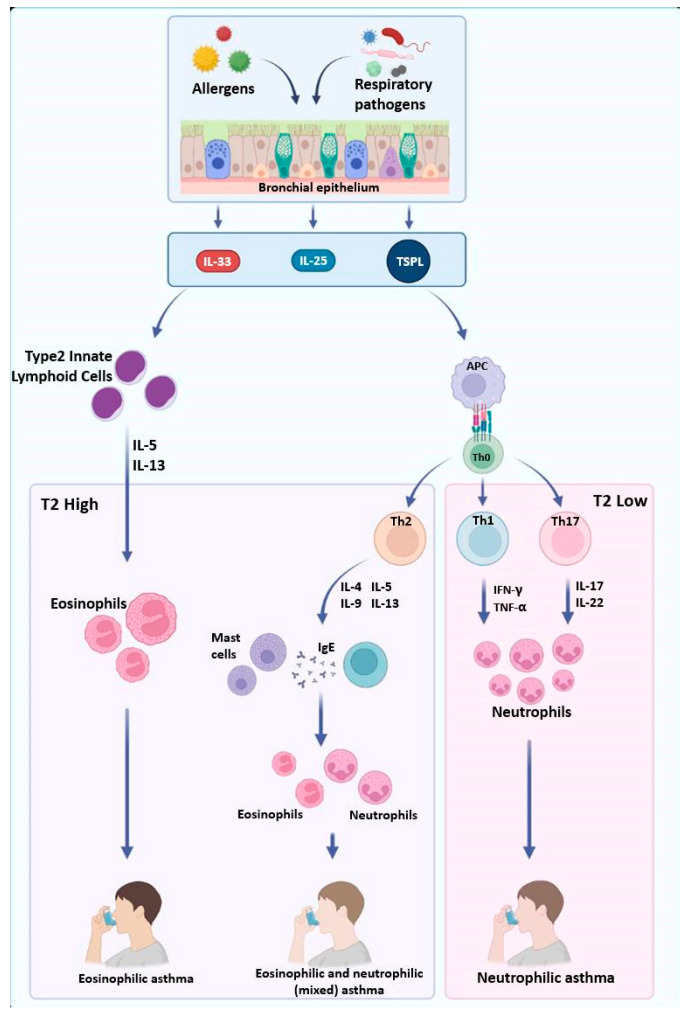
**Immunopathological pathways driving asthma endotypes.** This figure illustrates the immunopathological mechanisms of asthma, highlighting the role of epithelial-derived cytokines in driving different inflammatory phenotypes. Allergens and respiratory pathogens stimulate bronchial epithelial cells to release IL-33, IL-25, and TSLP, which activate type 2 innate lymphoid cells (ILC2) and antigen-presenting cells (APCs). A T2-high response leads to eosinophilic inflammation via Th2 cytokines (IL-4, IL-5, IL-9, and IL-13), contributing to eosinophilic or mixed asthma. In contrast, a T2-low response involves Th1 and Th17 activation, promoting neutrophilic inflammation through IFN-γ, TNF-α, IL-17, and IL-22, characteristic of neutrophilic asthma. **Source:** created with Biorender.

Personalized treatments targeting the molecular and inflammatory phenotypes of this heterogeneous endotype might offer new ways of improving outcomes in patients with severe non-eosinophilic asthma. The immunological features of T2-low asthma and its divergence from T2-high mechanisms are illustrated in Figure 1 and Table 1.

## 3. Role of Pathogens in Asthma Exacerbation

Respiratory infections are a major driver of asthma exacerbations, particularly in the pediatric population. Increasing evidence demonstrates that common respiratory viruses (e.g., respiratory syncytial virus (RSV), rhinovirus (RV), influenza virus, and SARS-CoV-2) and certain microbial exposures (e.g., *Aspergillus fumigatus*, and probiotics) can profoundly influence host immune responses via epigenetic remodeling, defined as heritable but reversible changes in gene expression regulated through DNA methylation, histone modifications, and non-coding RNAs without altering the DNA sequence. These changes not only modulate susceptibility to asthma but also affect disease severity and therapeutic responsiveness.

### 3.1. Respiratory Viral Pathogens

**Figure 2:**
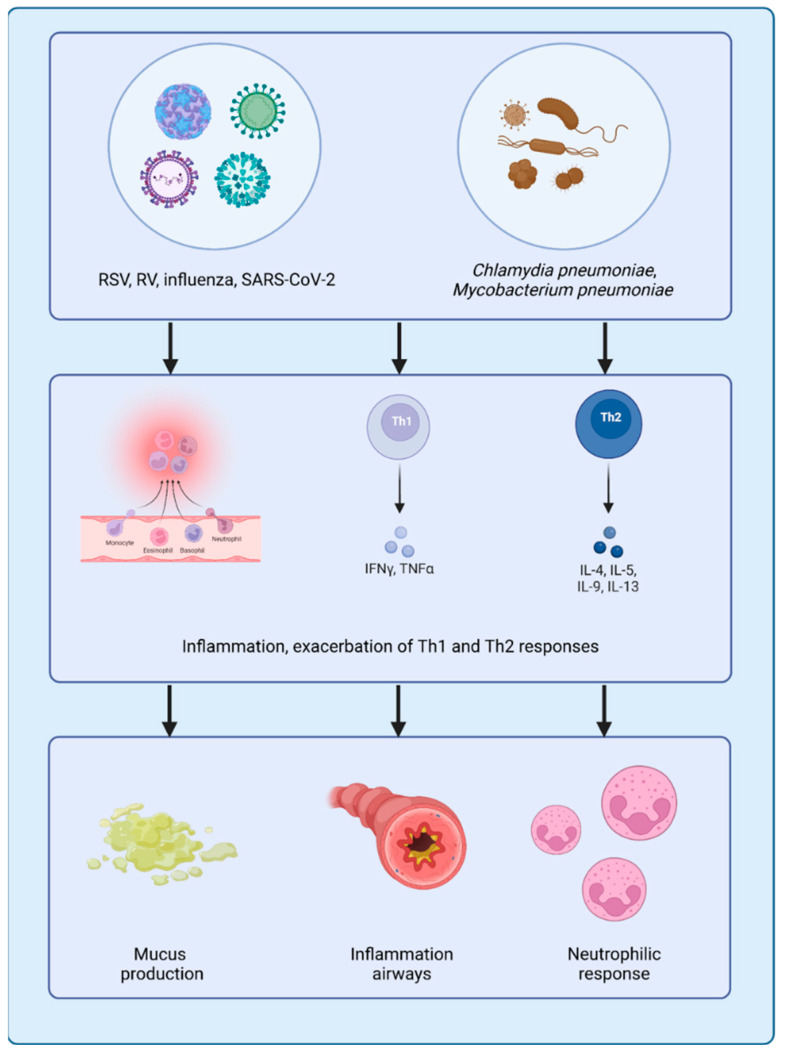
**Impact of viral and bacterial infections on asthma pathophysiology.** Pathogen-mediated inflammation exacerbates asthma-related symptoms. Both viral and bacterial pathogens can trigger inflammation and amplify Th1- and Th2-mediated immune responses. In the context of asthma, augmented inflammation contributes to increased mucus production, airway hyperresponsiveness, and airway inflammation, which is often accompanied by a neutrophilic or eosinophilic immune response, depending on the type and severity of the exacerbation. **Source:** created with Biorender.

Respiratory viruses represent potent environmental triggers that interact dynamically with the host epigenome, shaping immune responses and influencing the occurrence and severity of asthma exacerbation in children (Figure 2) [13].

The above-mentioned pathogens, most notably RSV and human RV, are able to induce the host immune response not only through direct inflammation but also by inducing long-lasting epigenetic modifications. Such modifications, like DNA methylation and histone modifications, may impact asthma predisposition, severity, and responsiveness to treatment [14,59]. Among the various epigenetic mechanisms, DNA methylation modifications are the most robustly supported by pediatric studies, particularly those implicating ORMDL3 and GSDMB in asthma susceptibility and inflammation. These epigenetic modifications can lead to long-term effects on immune function, sustaining airway inflammation and hyperresponsiveness in the absence of active infection. Respiratory pathogens, particularly when encountered during early childhood, are among the most impactful environmental factors contributing to the onset and exacerbation of pediatric asthma. Viruses, bacteria, and fungi are capable of altering the immune homeostasis, with a persistent inflamed state in the airways. The host immune response to these infections, particularly in early life, can lead to persistent inflammatory changes in the airways, contributing to the development and exacerbation of asthma [60].

The mechanisms by which respiratory pathogens exacerbate asthma are complex. Viral respiratory infections, especially RSV and RV, have long been associated with asthma exacerbation. These viruses can damage airway epithelium, promote mucus hypersecretion, and increase airway hyperresponsiveness [61]. Concomitantly, they activate immune responses that involve the release of proinflammatory cytokines, such as IL-4, IL-5, and IL-13, which stimulate Th2-mediated inflammation, a key characteristic of allergic asthma. RSV is a major cause of severe lower respiratory infection in infants and is strongly associated with asthma development. Early-life RSV infections are known to induce lasting epigenetic modifications, including altered DNA methylation, which can exacerbate airway inflammation and increase asthma risk [62]. Furthermore, RSV induces the expression of proinflammatory microRNAs like miR-21, which promote inflammation, contributing to persistent airway hyperreactivity [63,64]. RV, the main cause of the common cold, has been strongly associated with asthma exacerbations in children. RV infection may be able to alter the epigenetic profiles in airway epithelial cells, driving persistent inflammation and airway remodeling. Early-life RV infection has effects that are particularly strong in atopic children and confers a marked risk of subsequent asthma development [13,65]. A birth cohort study identified an association between variants in the chromosome 17q21 locus and RV-related, but not RSV-related, wheezing illnesses.

Notably, the expression of *ORMDL3* (encoding ORMDL sphingolipid biosynthesis regulator 3) and *GSDMB* (encoding gasdermin B), both asthma-associated genes located in the 17q21 locus, was significantly elevated in RV-stimulated peripheral blood mononuclear cells (PBMCs) compared to unstimulated PBMCs [66].

Further investigation showed hypomethylation in the 5′-untranslated region (5′-UTR) of the *ORMDL3* gene specifically in CD8^+^ T cells, but not in other T cell subsets. This finding suggests that DNA methylation differences at this locus may enhance T cell-mediated inflammation. The combination of increased ORMDL3 mRNA expression and altered methylation in CD8+ T cells may predispose individuals to an exaggerated inflammatory response following RV infection. These epigenetic mechanisms provide a potential explanation for the observed association between ORMDL3 risk alleles and RV-induced wheezing and early-onset asthma phenotypes [67]. In addition, RV-induced wheezing during early childhood has been linked to epigenetic changes at multiple asthma susceptibility loci. These include the promoter region of the *SMAD3* gene (chromosome 15q22.33), as well as intronic regions within the DDO (D-aspartate oxidase) and *METTL24* (methyltransferase-like 24) genes at chromosome 6q21 [68].

Another study demonstrated that an IFN-γ-enriched microenvironment enhances the resistance of the respiratory epithelium to RSV infection, potentially through epigenetic modification of *DDX58*, which encodes the DExD/H-box helicase 58. This modification led to increased DDX58 expression and augmented antiviral defense mechanisms [15]. RSV infection has also been shown to upregulate lysine demethylase 5B, a histone H3K4 demethylase, in dendritic cells. Experimental inhibition of KDM5B activity shifted immune responses toward a Th1 phenotype while suppressing Th2 polarization, highlighting the enzyme’s role in maintaining the Th2-skewed inflammation characteristic of allergic asthma [69]. Furthermore, in airway epithelial cells, RSV’s non-structural protein 1 (NS1) was found to interact with histone H2BD, inducing its ubiquitination. This epigenetic modification triggered the activation of *HOX* gene expression, which is involved in embryonic patterning and lung morphogenesis. Aberrant HOX activation in this context may contribute to abnormal lung development and heightened asthma susceptibility in children following early-life RSV infections [70].

Other respiratory viruses also play a role in asthma pathophysiology. Among them, the influenza virus is one of the most well-characterized contributors, frequently associated with severe asthma exacerbations. Influenza infection elicits a strong proinflammatory response in the airways, leading to increased production of cytokines that exacerbate bronchial hyperresponsiveness, mucus secretion, and airway obstruction. These effects are particularly pronounced in children, who are more susceptible to virus-induced asthma worsening [71,72]. Due to these risks, annual influenza vaccination is strongly recommended for children and adults with asthma as a preventive measure to reduce the frequency and severity of exacerbations [73].

Similarly, human metapneumovirus (hMPV) is a leading cause of respiratory tract infections in young children and has been implicated in asthma exacerbations. hMPV-associated airway inflammation results in increased mucus production and enhanced bronchial reactivity, resulting in difficulties with asthma control and risk for hospitalization [74,75]. This virus has common pathogenicity with parainfluenza viruses, which cause disorders such as croup and bronchitis, among others. In asthmatic individuals, parainfluenza virus infections exacerbate airway inflammation and obstruction, contributing to wheezing and respiratory distress and further increasing the overall disease burden [76].

The strongest evidence for virus-induced epigenetic modulation in pediatric asthma comes from studies on *respiratory syncytial virus* (*RSV*) and *rhinovirus* (*RV*). RSV has been linked to changes in DNA methylation patterns, upregulation of proinflammatory miRNAs (e.g., miR-21), and histone alterations in airway epithelial and immune cells. RV infection is associated with altered methylation at the ORMDL3 gene locus and miR-155 upregulation in asthmatic children, especially those with atopy.

Beyond paramyxoviruses, adenoviruses have also emerged as important contributors to asthma pathogenesis. In addition to causing severe lower respiratory tract infections, adenoviral infections are capable of inducing long-term epigenetic modifications in airway epithelial cells, as widely demonstrated by in vitro experiments [77].

Adenovirus infection has been reported to modulate multiple epigenetic mechanisms, in particular altering histone modification and interfering with transcriptional regulation in host cells. One of the most prominent viral effectors, the E1A protein, mediates acetylation of histone H3 lysines, in particular H3K9 and H3K18, and is associated with the modulation of various transcription factors that affect gene expression profiles [78,79]. Another protein, adenoviral protein VII, is produced in the late phase of infection and is transported into the nucleus, associates with host chromatin, and modifies its composition. Similarly, the adenoviral E1A protein localizes to host chromatin, modulates histone modifications, and induces widespread transcriptional reprogramming in infected cells [80,81]. The virus-induced epigenetic alteration can sustain proinflammatory conditions in the airways and enhance the risk of developing asthma, particularly in children with a prior history of respiratory dysfunction [82]. The role of coronaviruses, particularly SARS-CoV-2, in asthma exacerbations has also garnered considerable interest during the COVID-19 pandemic. It modulates the host epigenome to enhance its infectivity and immune evasion, partly by impairing the innate immune response. A critical factor in this interaction is the angiotensin-converting enzyme 2 (ACE2) receptor, whose expression is markedly upregulated in infected airway epithelial cells. This increased ACE2 expression has been linked to epigenetic changes, including hypomethylation near its transcription start site. Moreover, the virus promotes an active chromatin landscape through histone modifications, particularly H3K4me1, H3K4me3, and H3K27Ac, which are associated with gene activation and enhanced viral entry [83].

Although the exact association between COVID-19 and asthma is still under investigation, it is well known that coronaviruses induce a significant inflammatory response that potentially increases the risk of exacerbations in susceptible individuals [84,85,86,87]. Overall, these results demonstrate that respiratory viruses are not only transient triggers of asthma but active modulators of disease progression. These pathogens contribute to the chronicity and severity of asthma by inducing inflammation and epigenetic modification and disrupting epithelial integrity. The emerging evidence highlighted the necessity of innovative target therapy that addresses not only the acute viral infections but also long-term molecular consequences, offering a more comprehensive approach to pediatric asthma management.

Table 2 summarizes the main respiratory viruses implicated in pediatric asthma, highlighting the associated epigenetic modifications, target genes, and functional consequences in airway inflammation and remodeling.

### 3.2. Bacterial Pathogens

Bacterial infections can significantly contribute to asthma exacerbations, eliciting immune responses that sustain chronic airway inflammation. Among respiratory pathogens, *Mycoplasma pneumoniae* and *Chlamydia pneumoniae*, often classified as atypical bacteria, have been frequently associated with both the development and worsening of asthma, particularly in children. These bacteria are not only implicated in acute respiratory infections but are also capable of inducing long-term inflammatory changes, even after clinical resolution of the infection [88]. *M. pneumoniae*, in particular, has been linked to the initiation and exacerbation of asthma. It promotes sustained airway inflammation by activating multiple immune pathways, leading to the release of proinflammatory cytokines such as TNF-α and IL-6. This cytokine cascade contributes to airway hyperresponsiveness, wheezing, and chronic inflammation, hallmarks of asthma exacerbation [89]. Similarly, *C. pneumoniae* has been associated with both acute flare-ups and persistent asthma symptoms. This pathogen tends to skew the immune response toward a neutrophilic profile, which is often linked to more severe and corticosteroid-resistant asthma phenotypes [90].

In addition to atypical bacteria, *Klebsiella pneumoniae* has emerged as a relevant bacterial pathogen in asthma exacerbations, particularly among individuals with pre-existing lung conditions. *K. pneumoniae* is a gram-negative bacterium commonly associated with severe respiratory tract infections, including pneumonia [91,92]. In asthmatic individuals, infection with *K. pneumoniae* can elicit a robust inflammatory response, characterized by mucus overproduction and enhanced airway hyperresponsiveness. This inflammation is often mediated by Th2 immune activation, with elevated production of IL-4 and IL-13, cytokines known to contribute to goblet cell hyperplasia and excessive mucus secretions [93,94,95,96]. Animal studies have further demonstrated that *K. pneumoniae* infection can aggravate asthma by inducing both neutrophilic and eosinophilic inflammation, worsening airway obstruction, and increasing the frequency and severity of asthma attacks [97].

Alterations in the host microbiome have been implicated in asthma pathophysiology. In a murine model, perinatal exposure to vancomycin led to increased susceptibility to allergic asthma, accompanied by airway inflammation, elevated serum IgE, and a reduction in regulatory T cells [98]. Although the specific microbial taxa affected were not definitely identified, the authors noted a probable reduction in *Clostridioides* species, consistent with findings from other studies [98,99].

Overall, bacterial pathogens contribute to asthma exacerbation through several mechanisms, including immune dysregulation, mucus hypersecretion, and chronic airway inflammation. In particular, the shift from an eosinophilic to a neutrophilic inflammatory profile could make asthma more refractory to corticosteroid therapy. Thus, understanding the role of these bacterial infections in asthma is crucial for the development of more effective treatment strategies, particularly for managing severe and steroid-resistant asthma.

### 3.3. Fungal Pathogens

While most of the data on the epigenetic modulation induced by fungi stem from animal models, direct evidence in humans is limited. Observational associations in pediatric studies suggest potential relevance, but epigenetic mechanisms remain underexplored in human studies. Fungal pathogens, especially *Aspergillus fumigatus*, are highly involved in asthma exacerbations and impact children with allergic bronchopulmonary aspergillosis (ABPA). ABPA is an allergic condition characterized by hypersensitivity to *A. fumigatus*, leading to severe airway inflammation, mucus overproduction, and persistent bronchial obstruction. Inhalation of fungal spores can initiate and perpetuate an allergic immune response, leading to airway hyperreactivity and severe asthma symptoms [100]. Nonetheless, many studies have proven that children with asthma who acquire ABPA experience more recurrent and severe acute exacerbations due to the enhancement of the Th2 immune response. These responses include elevated Th2 cytokines, namely, IL-4 and IL-13, which induce IgE production and eosinophilic inflammation.

Elevated IgE levels are a hallmark of ABPA and other fungal sensitizations, as they amplify allergic inflammation, leading to increased bronchial obstruction, wheezing, and difficulty in controlling asthma symptoms [101]. In sensitized individuals, exposure to *A. fumigatus* spores can trigger an inflammatory cascade involving the release of histamines, leukotrienes, and other mediators. This process not only worsens asthma but also contributes to airway remodeling over time, which can lead to long-term complications such as bronchiectasis if not adequately managed. The combination of inflammation, mucus plugging, and structural airway changes poses significant challenges in asthma management for children with ABPA [100,101]. Interestingly, animal studies suggest a possible epigenetic component in fungal-induced immune modulation. In one study, maternal exposure to *A. fumigatus* during late gestation was associated with reduced IgE levels and pulmonary eosinophilia in offspring. These protective effects were linked to changes in CpG methylation at the promoter regions of IFN-γ and IL-4 genes, suggesting that fungal exposure may influence asthma risk through epigenetic reprogramming [102]. However, to date, these findings have not been validated in established asthma animal models, and their clinical relevance remains to be clarified.

Other fungi may also contribute to asthma exacerbation. Early-life gut colonization by *Candida* and *Rhodotorula* species has been associated with an increased risk of atopy and childhood asthma, potentially via alterations in host immune development and tolerance [103].

### 3.4. Coinfections

Pathogen respiratory coinfections are common in children [104,105]. Although the interaction between multiple pathogens and host cells during early life may disrupt transcriptional programmes, potentially through epigenetic mechanisms and impaired immune response [106], the exact role of coinfections in shaping airway epigenetics in children remains unclear and requires further investigation.

#### 3.4.1. Coinfections Involving Viruses and Pathogenic Bacteria

Several opportunistic bacterial pathogens have been linked to illnesses associated with RSV infection and pneumonia, including *K. pneumoniae*, *Moraxella catarrhalis*, and *Haemophilus influenzae*. Evidence suggests that RSV may facilitate bacterial colonization and intensify disease severity caused by these pathogens. For example, *K. pneumoniae* has been frequently detected in RSV-positive samples, though prevalence rates vary across studies. One study reported *K. pneumoniae* in 66% of RSV cases and 58% of RSV-associated pneumonia cases [107], while another study observed a lower coinfection rate [108]. Coinfection with RSV and *K. pneumoniae* has been associated with more severe clinical outcomes, such as increased oxygen requirements, elevated C-reactive protein levels, and a higher risk of recurrent wheezing in children under three years old [108]. Furthermore, the concurrent presence of *K. pneumoniae*, *M. catarrhalis*, and *H. influenzae* in RSV-positive samples has been correlated with an increased likelihood of developing asthma, especially mild to moderate forms [109]. The mechanisms driving these coinfections are multifaceted. RSV is thought to promote bacterial colonization by skewing the immune response toward a Th2 phenotype [93,110], which in turn suppresses Th17 polarization, a pathway essential for neutrophil recruitment and effective bacterial clearance [95,111]. However, the findings are not entirely consistent. For instance, in models of allergic sensitization, inducing a Th2 response did not significantly affect the burden of *K. pneumoniae* [97], suggesting that RSV’s role in facilitating bacterial coinfection may depend on additional host or environmental factors. Understanding the interplay between RSV and bacterial pathogens like *K. pneumoniae*, *M. catarrhalis*, and *H. influenzae* is crucial for developing more targeted prevention and treatment strategies for pediatric respiratory infections.

Similar protective effects were observed in house dust mite (HDM)-sensitized mice, which also showed lower bacterial loads and mortality rates during influenza/*S. pneumoniae* coinfection [112].

Animal studies provide an additional layer of understanding. In a murine model of ovalbumin-sensitized allergic airway disease (AAD), sensitized mice demonstrated improved bacterial clearance and survival following coinfection with influenza and *Streptococcus pneumoniae* compared to non-sensitized mice. Similar results were observed in house dust mite (HDM)-sensitized mice, which also showed lower bacterial loads and mortality rates during influenza/*S. pneumoniae* coinfection. This protection appeared to be mediated by elevated levels of TGFβ, which were already increased before influenza infection. When TGFβ receptor II (TGFβRII) was deleted in sensitized mice, the protective effect was lost, highlighting the critical role of TGFβ in host defence [112]. Since TGFβ is often upregulated in individuals with asthma, it may similarly contribute to protection against viral and bacterial coinfections in humans with asthma [113]. Independent findings in a model of *Aspergillus fumigatus*-sensitized AAD also support this hypothesis. In these mice, sensitization led to reduced bacterial burden and mortality during influenza/*S. pneumoniae* coinfection compared to non-sensitized controls [114]. As type I and III interferons are central to regulating susceptibility to secondary bacterial infections following influenza, it is likely that asthma-related immune changes influence these interferon pathways. However, neither of the studies reported measurements of interferon levels, leaving their precise contribution to this protective mechanism unresolved.

#### 3.4.2. Coinfections Involving Different Respiratory Viruses

Coinfections involving RSV and other respiratory viruses are commonly observed, particularly in infants, and are known to influence both asthma development and severity. Several viruses frequently coinfect with RSV, including RV, influenza A virus, human MPV, and parainfluenza viruses [115,116,117]. Among these, RV is a major contributor to asthma exacerbations and long-term wheezing disorders, with the risk further elevated when coinfection with RSV occurs. Although RV typically causes mild upper respiratory infections, its coinfection with RSV has been linked to severe lower respiratory tract infections (LRTIs) [118,119]. Evidence suggests that RSV–RV coinfections predispose children to recurrent bronchiolar obstruction, wheezing, and allergic sensitization, all hallmarks of asthma development. One study showed that approximately 83% of children aged 6 to 8 years who experienced bronchiolitis due to coinfections developed recurrent wheezing, compared to 70% of children with bronchiolitis caused by a single pathogen. Moreover, the same study reported that hospitalization rates were twice as high in children with coinfections versus those with single infections [120]. This heightened risk may be influenced by genetic predispositions, including a family history of asthma or other atopic conditions such as allergic rhinitis and atopic dermatitis.

Although the precise mechanism remains to be fully understood, interactions between RSV and RV seem to disrupt airway immune responses, possibly by amplifying inflammatory cytokines that promote a Th2-skewed immune profile, closely associated with both asthma and other allergic diseases [119]. Interestingly, RV causes less damage to airway epithelial integrity compared to RSV, suggesting that the sequence of infections may also contribute to the severity of disease outcomes.

Influenza A virus is another key respiratory pathogen that frequently coinfects with RSV, largely due to their overlapping seasonal patterns [121,122]. RSV-influenza coinfections are often associated with more severe clinical outcomes, including prolonged hospitalization, Intensive Care Unit (ICU) admission, and, in some cases, fatal outcomes [123,124]. Although influenza is less directly associated with asthma compared to RV, coinfections with RSV have been reported to increase the risk of long-term respiratory complications, such as persistent wheezing and potentially asthma development.

One proposed mechanism involves the suppression of IFN-γ-producing T cells during influenza–RSV coinfections, leading to impaired antiviral responses. This immune modulation may exacerbate airway inflammation and contribute to airway remodeling, both of which are critical factors in asthma pathogenesis [125]. Interestingly, animal and in vitro studies have shown that influenza can inhibit RSV replication through the upregulation of interferon-stimulated genes (ISGs), such as IFIT1-3 and IFI44, which interfere with RSV gene expression [126]. While this interaction may reduce the total viral load during coinfection, the resulting airway inflammation can still promote conditions conducive to asthma development.

hMPV, which shares many clinical and molecular features with RSV, is another significant cause of acute respiratory tract infections (ARTIs) [127]. Coinfections involving RSV and hMPV are associated with increased disease severity, including higher rates of ICU admission and prolonged hospital stays [128]. However, the impact of RSV–hMPV coinfections on asthma development remains less clear. Reports suggest that younger children, particularly those under six months of age, are more susceptible to severe illness during RSV–hMPV coinfections, potentially due to immature immune responses [129,130].

The interaction between these two viruses may amplify airway inflammation, thereby increasing the risk of wheezing and asthma-like symptoms. Although RSV tends to dominate in such coinfections by impairing hMPV replication through interferon pathways [131], the overall inflammatory burden from dual infections may nonetheless predispose to asthma-like outcomes.

## 4. Epigenetic Mechanisms in Asthma

Epigenetic mechanisms refer to inheritable, yet dynamic and reversible, changes in gene expression that occur without altering the underlying DNA sequence [38,132]. These modifications influence how genes are expressed and play a crucial role in both the development and exacerbation of asthma. One key epigenetic process is DNA methylation, which involves the addition of a methyl group to the cytosine base, particularly within CpG islands in gene promoter regions. This modification typically silences gene expression by preventing transcription factors from binding to the DNA [133]. Importantly, DNA methylation patterns are tissue-specific, meaning their effects can differ significantly between immune cells and airway epithelial cells [134]. In asthma, abnormal DNA methylation patterns have been closely linked to disease pathogenesis. For instance, hypermethylation of genes involved in anti-inflammatory responses, such as *IFN-γ*, *LAT*, and *HDAC2*, can suppress the expression of protective genes, thereby worsening airway inflammation [135]. Conversely, hypomethylation of proinflammatory genes, including *iNOS*, *IL-13*, and *CXCL8*, can promote their overexpression, further amplifying airway inflammation and asthma severity [135]. Environmental factors like air pollution, diet, and allergens can alter these epigenetic patterns, contributing to the chronic nature and progression of asthma [12,136]. Recent research has identified distinct DNA methylation markers in both bronchial epithelial cells and peripheral blood mononuclear cells (PBMCs), highlighting significant differences between asthmatics and non-asthmatics [137]. Importantly, recent studies have demonstrated that DNA methylation patterns found in easily accessible tissues, such as nasal epithelial cells, can provide valuable insights into pediatric asthma and may serve as non-invasive biomarkers for diagnosis and endotype stratification. For example, methylation signatures at *IL13*, *POSTN*, and *CLCA1* loci in nasal epithelium have been associated with Th2-high asthma phenotypes and linked to disease severity [138]. Additionally, hypomethylation at the *ORMDL3* and *GSDMB* loci within the 17q21 asthma susceptibility region has been observed in children with early-onset asthma and rhinovirus-related wheezing, underscoring their potential in risk prediction and personalized treatment planning [16]. These findings are further supported by transcriptomic profiling studies highlighting immune diversity in pediatric asthma endotypes [139].

Finally, obesity-associated pediatric asthma displays a distinct epigenetic profile. Peripheral blood mononuclear cells show hypomethylation of Th1/monocyte-related genes (e.g., CCL5, IL2RA, and TBX21) and hypermethylation of FCER2 and TGFB1, along with upregulation of CDC42-related pathways, supporting a non-T2/Th1-skewed, immunometabolic phenotype [139,140].

### 4.1. Histones

Histones are proteins that help to organize DNA into chromatin, and their post-translational modifications, such as acetylation and methylation, influence gene expression. Histone acetylation, mediated by histone acetyltransferases (HATs), typically promotes gene transcription, while histone deacetylation, mediated by histone deacetylases (HDACs), results in gene repression [141,142]. Histone modifications play a crucial role in regulating gene expression in T2-high asthma. Specifically, increased acetylation of histones at the promoter of proinflammatory genes can lead to their overexpression, driving persistent airway inflammation [12]. Conversely, reduced acetylation of anti-inflammatory genes silences these protective pathways, exacerbating asthma symptoms.

HDACs are classified into two main groups: class I (HDAC1–3 and 8), which are primarily localized in the nucleus, and class II (HDAC4–7, 9, and 10), which shuttle between the nucleus and cytoplasm in response to intracellular signalling [143,144]. These enzymes interact with corepressor molecules, facilitating gene repression and adding a layer of specificity by enabling certain HDACs to selectively regulate particular genes [145]. Among them, HDAC2 plays a pivotal role in regulating the expression of inflammatory genes. Notably, reduced HDAC2 levels have been linked to corticosteroid resistance in asthma patients, positioning HDAC2 as a promising therapeutic target [146].

Histone modifications are not only crucial for general gene regulation but also contribute to defining specific asthma endotypes. For instance, Th2-driven asthma, which is characterized by elevated levels of IL-4 and IL-13, is associated with histone hyperacetylation at H3K9 and trimethylation at H3K4 [147]. These epigenetic modifications enhance the transcription of cytokines that drive the Th2 immune response, thereby worsening asthma symptoms [148]. Furthermore, increased levels of key cytokines such as IFN-γ and IL-4 are linked to H3K4 trimethylation, reinforcing the link between histone modifications and the production of inflammatory cytokines [149].

In childhood asthma, specific epigenetic alterations, such as acetylation of the *Foxp3* [150] and *IL13* genes, have been shown to influence immune regulation, further linking early-life epigenetic modifications to an increased susceptibility to asthma [151]. Additionally, histone hyperacetylation of the ORMDL3 gene, which is involved in airway remodeling, has been directly associated with asthma development and with the structural changes observed in the airways of asthma patients [148,152]. This highlights the role of histone modifications not only in inflammation but also in the long-term airway remodeling that characterizes chronic asthma.

Moreover, histone modifications regulate the production of chemokines, which are essential in the recruitment and differentiation of Th2 cells in asthma. In particular, dimethylation of CCR4 and CCL5 has been associated with Th2-driven inflammation, promoting eosinophil recruitment and further exacerbating airway inflammation [153].

Given the central role of histone modifications in asthma pathogenesis, therapeutic strategies targeting these epigenetic changes are gaining considerable interest. Both HDAC inhibitors and HAT modulators are currently under investigation as potential treatments, particularly for patients with severe, steroid-resistant asthma.

### 4.2. Non-Coding RNAs

In addition to histone modifications, non-coding RNAs (ncRNAs) play a crucial role in regulating inflammation in asthma. Among these, ncRNAs, particularly microRNAs (miRNAs), are particularly important, as they control gene expression at the post-transcriptional level. miRNAs exert their effects by binding to messenger RNAs (mRNAs), leading to their degradation or inhibition of translation [154]. In asthma, dysregulation of several miRNAs contributes to the disease’s pathogenesis. For instance, miR-155 plays a pivotal role in promoting inflammation by targeting genes involved in anti-inflammatory pathways, thereby intensifying inflammatory responses [155,156], while miR-126 has been shown to suppress inflammation by targeting proinflammatory genes, thereby modulating immune responses and helping to reduce inflammation [154,157,158].

In children with RV infections, miR-155 is significantly elevated and correlates with more severe asthma symptoms. Its upregulation enhances both Th1 (antiviral) and Th2 (allergic) immune responses, contributing to immune dysregulation and increased inflammation. These findings suggest that targeting miR-155 may offer novel therapeutic strategies to reduce asthma severity and manage inflammatory responses in respiratory diseases [154,159].

In pediatric asthma, other miRNAs have also been identified as significant, including miR-21 and miR-146a. miR-21 is frequently elevated in asthma and contributes to airway inflammation and remodeling by targeting various anti-inflammatory and pro-apoptotic genes [160] such as PTEN (phosphatase and tensin homolog), a key negative regulator of inflammatory pathways [161]. By downregulating PTEN, miR-21 activates the PI3K/Akt signaling pathway, enhancing the survival of inflammatory cells like eosinophils and T cells in the airways [162]. miR-21 also plays a crucial role in airway remodeling. By regulating the expression of Transforming Growth Factor-beta (TGF-β), miR-21 promotes fibroblast activation and the differentiation of myofibroblasts, leading to collagen deposition and airway fibrosis. These structural changes contribute to airway narrowing and progressive decline in lung function [163]. Elevated levels of miR-21 have also been linked to glucocorticoid resistance in severe asthma, making inflammation more challenging to manage with standard steroid therapies [164].

Concerning miR-146a, it modulates the immune response by targeting genes involved in the NF-kB signaling pathway, a key driver of inflammation. By targeting TRAF6 and IRAK1, miR-146a downregulates NF-κB activation, thus preventing excessive inflammation in the airways [165,166,167]. This is particularly important in pediatric asthma, where chronic inflammation can lead to long-term lung damage. Additionally, miR-146a influences various immune cell functions, including those of macrophages and dendritic cells. It promotes a regulatory T cell (Treg) phenotype, which helps to maintain immune tolerance and modulate the Th2 response, thereby reducing airway hyperreactivity and inflammation [165,168].

Given their key roles in asthma pathogenesis, these miRNAs are emerging as promising biomarkers for both asthma diagnosis and treatment. Therapies that target miRNAs, either through miRNA mimics or inhibitors, are currently under investigation as potential strategies for asthma treatment, particularly within the scope of personalized medicine [157,169] (Table 3).

Beyond miRNAs, extracellular vesicles (EVs) function as key carriers of miRNAs, proteins and lipids between airway epithelial/immune cells and microbes; their cargo and release are altered in asthma (e.g., IL-13-induced epithelial EVs and higher serum EV miR-125b/miR-126), and blocking exosome biogenesis mitigates experimental airway inflammation, underscoring diagnostic and therapeutic potential [170].

## 5. Novel Therapeutic Approaches

### 5.1. Epigenetic Modifications

The potential to target epigenetic modifications for asthma treatment represents an exciting and rapidly evolving area of research, largely due to the reversible nature of these modifications. Epigenetic changes can alter gene expression without modifying the underlying DNA sequence, making them particularly attractive therapeutic targets. Genome-wide association studies (GWAS) have contributed significantly to this field by identifying both genetic and epigenetic markers that could inform the development of novel asthma therapies [171]. This is especially relevant in childhood asthma, where early environmental exposures can induce lasting epigenetic changes that shape disease progression over time [172]. These insights provide new possibilities for managing asthma, particularly through interventions tailored to specific asthma endotypes. Since epigenetic modifications contribute to the heterogeneity observed across different asthma phenotypes, they present a valuable target for personalized therapeutic strategies. By addressing the epigenetic drivers of disease, therapies can be designed to target the underlying molecular mechanisms that contribute to asthma in specific patient populations, improving treatment efficacy while minimizing side effects.

### 5.2. DNA Methylation

One promising therapeutic approach involves targeting DNA methylation, a process in which methyl groups are added to DNA, often resulting in gene silencing. This mechanism plays a role in the pathogenesis of asthma by silencing genes that regulate inflammation. In asthma, particularly in T2-high asthma, hypermethylation of key anti-inflammatory genes has been linked to increased inflammation and poor disease control [173]. DNA methyltransferase inhibitors (DNMTi), such as 5-azacytidine, block the action of DNA methyltransferases (DNMTs) [174], which are responsible for adding methyl groups to DNA, thereby reversing the silencing of important anti-inflammatory genes [175,176].

Recent studies have demonstrated that small-molecule inhibitors of DNA methyltransferases, such as 5-azacytidine, can reverse aberrant methylation and restore the expression of silenced anti-inflammatory genes in both in vitro and in vivo models of allergic airway disease [177,178]. Preclinical studies have shown that 5-azacytidine can effectively reverse the hypermethylation of these genes, potentially restoring a balanced immune response and reducing chronic airway inflammation in asthma [179]. This strategy is particularly promising for T2-high asthma, a subtype driven by heightened sensitivity to Th2 cytokines like IL-4 and IL-13 [180]. By reactivating silenced regulatory genes, DNA methylation inhibitors may offer a novel and targeted means of controlling inflammation in patients with this asthma phenotype.

It is important to note that DNMT inhibitors, such as 5-azacytidine, are currently not approved for asthma treatment. Their application remains experimental, primarily based on preclinical studies in animal models. Moreover, these agents can exhibit off-target effects, including global hypomethylation, which may raise safety concerns such as genomic instability or unintended immune modulation.

Another promising strategy is the use of HDAC inhibitors. Histone acetylation facilitates a more relaxed chromatin structure, allowing gene transcription to occur. HDACs remove acetyl groups from histones, resulting in chromatin condensation and gene silencing. In asthma, this process can contribute to the suppression of genes involved in controlling inflammation [145].

HDAC inhibitors, such as vorinostat (SAHA), prevent the removal of acetyl groups, thereby promoting gene expression and reducing inflammation [181]. Research has shown that HDAC inhibitors can attenuate airway inflammation and improve lung function in animal models of asthma, with particularly encouraging results seen in T2-low and non-T2 asthma phenotypes, which are often less responsive to conventional corticosteroid therapies [145]. These subtypes often exhibit poor responsiveness to conventional corticosteroid therapies, highlighting the need for alternative treatment options.

As such, HDAC inhibitors represent a promising therapeutic strategy for asthma patients who do not respond adequately to standard treatments. By offering a more targeted and phenotype-specific approach, HDAC inhibitors could help address the unmet clinical needs of patients with difficult-to-treat asthma subtypes.

### 5.3. miRNA-Based Therapies

miRNA-based therapies represent another innovative therapeutic avenue for asthma management. miRNAs are small non-coding RNA molecules that regulate gene expression post-transcriptionally by binding to messenger RNAs (mRNAs), thereby preventing their translation into proteins. In asthma, specific miRNAs become dysregulated, contributing to chronic inflammation and disease exacerbations. Notably, miR-21 and miR-146a are among the miRNAs most frequently implicated in asthma pathogenesis [12].

miR-21 is often upregulated in asthmatic patients and is strongly associated with increased inflammation, particularly following viral infections [182,183]. Therapeutic strategies involving miRNA mimics or inhibitors aim to normalize these dysregulated miRNA levels. In preclinical models, miR-21 inhibitors have demonstrated the ability to be effective, where they were able to reduce airway inflammation and improve lung function. Targeting miRNAs like miR-21 offers a direct means of modulating the molecular pathways that drive asthma, making this an appealing therapeutic option for patients whose disease is influenced by miRNA dysregulation [182]. Moreover, miR-21 inhibition could provide a strategy to prevent virus-induced asthma exacerbations, which remain a major cause of hospitalizations in asthmatic patients [63].

Recent research has also highlighted the role of miR-146a, which is implicated in modulating immune responses. miR-146a plays a role in regulating Toll-like receptor (TLR) signaling pathways, which are involved in immune responses against pathogens and allergens. Inhibiting miR-146a has been shown to reduce the production of inflammatory cytokines, particularly in T2-high asthma, where allergic responses are driven by Th2 cytokines [168]. This suggests that miR-146a inhibition may represent a valuable strategy for controlling excessive inflammation in this asthma phenotype.

Therefore, miRNA-based therapies show great potential for treating asthma by targeting key dysregulated miRNAs, such as miR-21 and miR-146a. These approaches aim to restore normal miRNA levels, with the potential to reduce airway inflammation, improve lung function, and prevent exacerbations, particularly those triggered by viral infections. Ongoing research is essential to further elucidate the roles of miRNAs in asthma pathogenesis, which could pave the way for more effective and personalized therapeutic strategies, ultimately improving outcomes for patients living with this chronic respiratory disease.

Despite the promise of miRNA-based therapies, several challenges remain. Delivery to airway tissues is complex due to rapid degradation by nucleases, off-target binding, and poor cellular uptake [184]. Furthermore, unintended modulation of non-target genes and immune activation represent important safety concerns that require further investigation.

To date, the majority of evidence for miRNA-based interventions derives from preclinical animal models. For instance, in a murine model of allergic asthma, administration of an antagomir targeting miR-21 resulted in significant reductions in eosinophilic airway inflammation, airway hyperresponsiveness, and airway remodeling [185,186]. Additionally, miR-21 knockout mice exposed to ovalbumin (OVA) exhibited attenuated Th2 cytokine production and decreased airway hyperreactivity [187].

Similarly, miR-146a modulation has shown promising results in vivo. In a mouse model of Th17-predominant severe asthma, delivery of miR-146a-3p agomir by inhalation led to a marked decrease in airway hyperresponsiveness, neutrophilic inflammation, mucus secretion, and Th17 cell infiltration [188].

However, clinical evidence in humans remains limited. While several clinical studies have observed dysregulated miR-21 levels in exosomes or serum of asthmatic patients, showing correlations with disease severity such as IgE, IL-4, and lung function [187], no clinical trials have yet tested miRNA-based therapeutics in asthma. Thus, the translational potential of these interventions remains to be established in human subjects.

### 5.4. Probiotics

**Figure 3:**
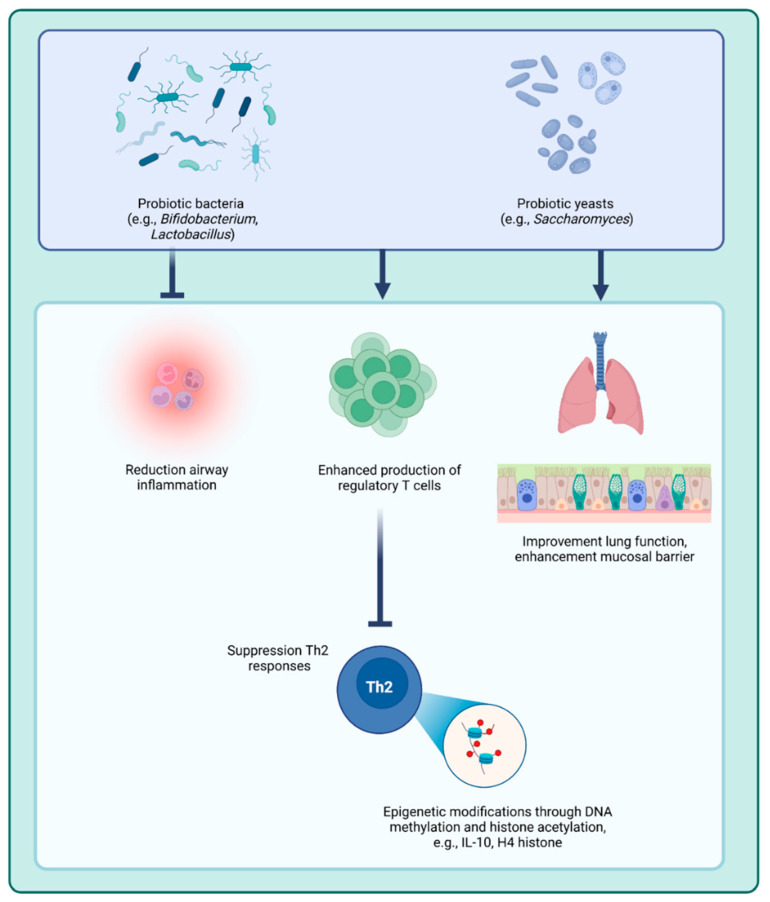
**Immunoepigenetic modulation of asthma by probiotic microorganisms.** Probiotic microorganisms contribute to a reduction in inflammation and subsequent asthma symptoms. Both probiotic bacteria and yeasts have been shown to reduce airway inflammation, improve lung function, and enhance the integrity of the mucosal barrier. These microorganisms modulate inflammation by promoting the production of regulatory T cells, which suppress Th2-mediated immune responses. This immunomodulation is partially mediated by epigenetic mechanisms, including DNA methylation at immune-related genetic loci (e.g., IL6 and IL10 genes) and histone acetylation, which collectively regulate the expression of proinflammatory and anti-inflammatory molecules. **Source:** created with Biorender.

Recent research has explored the relationship between probiotics and epigenetic modifications, highlighting their potential role in precision medicine approaches for asthma treatment. Probiotics, particularly strains of *Saccharomyces*, *Lactobacillus*, and *Bifidobacterium*, can modulate the gut microbiota, thereby influencing systemic immune responses through epigenetic mechanisms such as DNA methylation and histone modifications (Figure 3).

This modulation is crucial for developing personalized approaches to manage asthma and other immune-related diseases. Probiotics strains such as *Saccharomyces*, *Lactobacillus,* and *Bifidobacterium* have been shown to influence the balance between proinflammatory and anti-inflammatory immune responses in experimental models [189,190,191]. A study demonstrated that gut colonization by *Bifidobacterium*, *Akkermansia,* and *Faecalibacterium* species is associated with a lower incidence of asthma [103]. In asthma, where the immune system often skews toward a Th2-driven response (which drives allergic inflammation), probiotics may help restore immune balance by promoting a more regulated immune response and reducing inflammation. Probiotics have been shown to enhance the production of regulatory T cells, which suppress excessive Th2 activity and help maintain immune homeostasis. Additionally, probiotics may reduce airway inflammation and improve lung function by strengthening the mucosal barrier and limiting overactive immune responses to allergens [192,193].

Emerging evidence also suggests that early-life probiotic supplementation may reduce the risk of developing asthma and other allergic diseases. Probiotics appear to influence the gut–lung axis by modulating gut microbiota composition, which in turn shapes immune system development and reduces the likelihood of allergic sensitization [194]. By promoting a healthy gut microbiome, probiotics help foster immune tolerance, which may lower the risk of asthma exacerbations. Pre- and postnatal supplementation with *Lactobacillus reuteri* has been shown to induce beneficial epigenetic changes, such as modifying DNA methylation patterns in CD4^+^ helper T cells of infants and toddlers. These changes promote immune system maturation and reduce allergic responses [195]. Similarly, another study reported that supplementation with a combination of *Lactobacillus rhamnosus* GG and *Bifidobacterium lactis* during the pre- and postnatal periods resulted in global hypomethylation of immune-related genes, including *IL6R*, *IL5*, *CD38*, and *STAT3*, in treated children compared to untreated controls [196]. Probiotics may also influence epigenetic modifications through their impact on the gut–lung axis [197]. For example, they can modulate DNA methylation patterns, potentially reversing the hypermethylation of anti-inflammatory genes that is linked to increased inflammation in asthma. Additionally, probiotics may alter histone modifications, such as enhancing histone acetylation, which can upregulate the expression of anti-inflammatory genes and promote immune tolerance.

Several epigenetic mechanisms underlying asthma are influenced by environmental factors. Studies have shown that individuals living in rural environments—an effect often referred to as the “farming effect”—exhibit greater protection against asthma and other allergic diseases compared to those residing in urbanized, Westernized settings. This disparity is largely attributed to variations in environmental exposures, including greater microbial diversity and distinct lifestyle factors, which modulate immune system development and epigenetic regulatory pathways [198]. For example, one study reported that *Acinetobacter lwoffii*, a bacterium with probiotic properties, is more prevalent in a rural Russian community than in an adjacent urban Finnish population characterized by higher rates of childhood allergic diseases, including asthma [199]. Consistent with these findings, in a murine model of asthma, continuous intranasal exposure of pregnant mice to *A. lwoffii* resulted in offspring with reduced susceptibility to asthma. This protective effect was shown to be mediated via TLR-signaling, specifically involving TLR-2, TLR-3, TLR-4, TLR-7, and TLR-9 [200]. Further investigation revealed that part of this protection was attributable to the stabilization of histone H4 acetylation at the Interferon Gamma (IFNG) promoter in CD4+ T cells isolated from the spleens of the progeny [201]. Another study demonstrated that continuous intranasal exposure to *A. lwoffii* elicited a proinflammatory response that conferred protection against asthma in mice through an IL-6-dependent mechanism involving the epigenetic activation of IL-10 production [202].

In addition to naturally occurring strains, probiotics can be genetically modified to express immunostimulatory or immunoregulatory molecules, potentially offering an innovative strategy to reduce asthma severity [203,204,205].

Nevertheless, the results of the studies on probiotics in asthma, although promising, are currently inconclusive, and different studies have reported mixed outcomes. Thus, more studies are needed to determine the optimal strains, dosages, and timing of probiotic administration for consistent benefit in asthma prevention and management. It has previously been demonstrated that probiotics can be genetically modified to express immunostimulatory or immunoregulatory molecules, which may, in turn, reduce the severity of asthma. However, the completion of the findings is essential since the findings on probiotics in asthma are currently mixed. Numerous targeted studies are also needed in this area to select strains, dosing, and administration timing. Probiotics have the potential to affect epigenetic regulation and the principles of precision medicine, which involves selecting therapies based on the patient’s genetic, epigenetic, and microbiome profiles. The administration of probiotics, especially in the treatment of asthma in patients with epigenetic changes, which promote inflammation, can directly target the underlying molecular processes in asthma. Thus, using probiotics can help in the rapid development of personalized therapeutic strategies for asthma and other inflammatory conditions.

While genetically engineered probiotics represent an innovative strategy for asthma therapy, their clinical application poses significant regulatory and safety challenges. Concerns include horizontal gene transfer, unintended immune reactions, and long-term ecological impacts on the microbiota. Regulatory frameworks for live biotherapeutic products are still evolving, and rigorous preclinical and clinical assessments are needed to ensure biosafety, stability, and efficacy.

### 5.5. Personalized Medicine

One of the most exciting aspects of targeting epigenetic modifications is their potential to advance personalized medicine. Personalized medicine involves tailoring medical treatments to the unique characteristics of each patient, often based on genetic, epigenetic, or molecular profiling. In the context of asthma, this approach is gaining traction as researchers and clinicians increasingly recognize that asthma is a heterogeneous disease, with multiple endotypes, each driven by distinct molecular pathways. Recent advancements have significantly expanded our understanding of how epigenetic modifications interact with genetic predispositions to shape asthma phenotypes and influence treatment responses (Table 4).

The identification of epigenetic biomarkers specific to different asthma endotypes offers the potential to enable more tailored treatment strategies. By integrating epigenetic data with genetic information, researchers can improve the ability to predict treatment responses and develop more effective, individualized therapies [190]. The epigenetic profiling of asthma endotypes is essential for advancing personalized medicine. Understanding key epigenetic modifications, such as DNA methylation, histone modifications, and non-coding RNAs, which distinguish T2-high, T2-low, and non-T2 asthma, is critical to the design of targeted, more precise therapeutic approaches.

For instance, DNA methylation inhibitors, HDAC inhibitors, and miRNA-based therapies hold promise for treating specific asthma endotypes by directly targeting the underlying molecular mechanisms [38]. The analysis of DNA methylation patterns or miRNA expression profiles may help to identify patients who are most likely to benefit from therapies such as corticosteroids, DNMT inhibitors, HDAC inhibitors, or miRNA-based treatments [38].

CRISPR-Cas9 gene editing technology represents another significant advancement in the field of personalized medicine, with the potential to further advance research in this area. In the context of asthma, this possibility has the potential to entirely revolutionize asthma therapy by focusing on disease-specific genetic or epigenetic alterations and changing them directly. CRISPR-Cas9 is expected to be particularly beneficial for addressing the genetic basis of distinct asthma endotypes. This technology can be used to precisely regulate epigenetic marks, including DNA methylation sites or miRNA binding sites that guide the development of asthma. Although still at the experimental stage, CRISPR-based treatments are a possible direction for highly precise personalized treatments that may change the epigenetics of asthma permanently [206].

Furthermore, the broader application of personalized medicine in asthma relies on using these biomarkers to guide therapeutic decisions, ensuring that each patient receives the most appropriate treatment for their specific disease subtype. This approach could enhance treatment efficacy, minimize side effects, and improve overall disease management, particularly for patients with severe or steroid-resistant asthma.

## 6. Conclusions

Infections caused by RSV, RV, influenza, and other respiratory pathogens play a key role in triggering asthma exacerbations, especially in children. These pathogens can induce epigenetic changes that sustain airway inflammation and worsen asthma symptoms. Gaining a deeper understanding of how infections interact with epigenetic mechanisms is critical for developing personalized treatment strategies. Future research should prioritize the identification of specific epigenetic biomarkers capable of predicting asthma exacerbations and guiding the development of targeted therapies. Although targeting epigenetic modifications represents a promising therapeutic strategy, these approaches are currently experimental and not part of standard clinical care. Further preclinical and clinical studies are needed to assess their safety, efficacy, and long-term outcomes before clinical adoption.

Approaches such as DNA methylation inhibitors, HDAC inhibitors, miRNA-based therapies, and selected probiotics are all being explored for their potential to improve outcomes through personalized interventions. Moreover, the identification of epigenetic biomarkers associated with different asthma endotypes supports the move toward personalized medicine, allowing for tailored therapeutic strategies. Continued research and robust clinical trials will be essential to translating these advances into practical, effective treatments for patients living with asthma.

## Figures and Tables

**Table 1:** Asthma endotypes.

FEATURE	T2-HIGH ASTHMA	T2-LOW ASTHMA
**DOMINANT IMMUNE CELLS**	Th2, ILC2, eosinophils, mast cells, B cells	Th1, Th17, neutrophils, antigen-presenting cells (APCs)
**KEY CYTOKINES**	IL-4, IL-5, IL-13, IL-9	IFN-γ, IL-17, TNF-α
**ALARMINS INVOLVED**	TSLP, IL-33, IL-25 (epithelial-derived)	Minimal role
**INFLAMMATORY PATTERN**	Eosinophilia, elevated IgE and FeNO	Neutrophilia, normal/low FeNO
**RESPONSE TO CORTICOSTEROIDS**	Responsive	Poor responsive or absent
**STRUCTURAL ALTERATIONS**	Airway remodeling, epithelial hyperplasia, mucus overproduction	Persistent epithelial damage, remodeling, impaired repair

**Table 2:** Summary of virus-associated epigenetic modifications in pediatric asthma.

Virus	Epigenetic Modification	Genes Affected	Function/Effect
RSV	DNA methylation, histone modification, miRNA	DDX58, KDM5B, HOX, miR-21	↑ DDX58 → antiviral defense; ↓ KDM5B → shift to Th1; HOX activation → lung remodeling; miR-21 ↑ inflammation/fibrosis
Rhinovirus	DNA methylation, miRNA	ORMDL3, GSDMB, SMAD3, DDO, METTL24, miR-155	Hypomethylation → ↑ ORMDL3/GSDMB → inflammation; ↑ miR-155 → ↑ Th1/Th2; ↑ asthma severity
Influenza	Histone modification, ISG activation (indirect)	IFIT1–3, IFI44 (indirect); general chromatin structure	↑ cytokines → ↑ mucus/bronchial reactivity; ISG expression may inhibit RSV replication
Adenovirus	Histone acetylation, chromatin remodeling	Histone H3 (K9, K18), chromatin targets (via E1A, protein VII)	H3K9/K18 acetylation → transcriptional reprogramming; ↑ proinflammatory gene expression
SARS-CoV-2	DNA hypomethylation, histone modification	ACE2, H3K4me1, H3K4me3, H3K27Ac marks	Hypomethylation → ↑ ACE2; histone marks → ↑ ACE2 transcription → ↑ viral entry/inflammation
hMPV	Not specified	-	↑ mucus, bronchial reactivity, hospitalization risk
Parainfluenza	Not specified	-	↑ airway inflammation and obstruction

**Table 3:** Epigenetic modifications in pediatric asthma.

Modification Type	Endotype	Involved Gene(s)	Etiological Agent	Implications
DNA methylation	T2-high	ORMDL3, GSDMB	*Rhinovirus* (implicated)	↑ Inflammation via CD8+ T cells; ↑ Expression linked to asthma susceptibility (17q21 locus)
		Anti-inflammatory genes (unspecified)	-	Hypermethylation leads to gene silencing and poor disease control
		Th2-associated CpG islands (nasal, buccal)	-	Methylation markers in nasal epithelium and buccal cells linked to Th2 activation
		IL6R, IL5, CD38, STAT3	Probiotics (*L. rhamnosus* GG, *B. lactis*) (implicated)	Global hypomethylation in immune genes after prenatal/postnatal supplementation
		GATA3, IL4	-	Hypo/hypermethylation regulates Th2 immune response
		SMAD3, DDO, METTL24	*Rhinovirus* (implicated)	Asthma susceptibility, early wheezing
		-	*Rhodotorula* species (implicated)	Associated with higher risk of atopy and asthma in children
		-	*Candida* species (implicated)	Early gut colonization linked to increased atopy and asthma risk
		ACE2	SARS-CoV-2 (implicated)	Hypomethylation → ↑ ACE2 expression → ↑ viral entry and inflammation
	T2-low	IL17A, IFNG	-	Epigenetic regulation of Th1 and Th17 response in neutrophilic asthma
		CXCL8, CXCL1	-	Genes involved in neutrophil recruitment and steroid resistance
		IFN-γ, IL-4 promoters	*Aspergillus* (prenatal) (implicated)	CpG methylation linked to protective effect in offspring
Histone modification	T2-high	H3K4, CCR4, CCL5	-	Trimethylation and dimethylation promote Th2 inflammation
		Foxp3, IL13	-	Acetylation → immune regulation, ↑ cytokine expression
		H3K4me3, H3K9ac	-	Linked to expression of IL-4 and IFN-γ
		H3K4me1, H3K4me3, H3K27Ac	SARS-CoV-2 (implicated)	Active chromatin marks → ↑ ACE2 transcription
		Histone H3 (K9, K18)	*Adenoviruses* (implicated)	E1A protein alters H3 acetylation → transcriptional reprogramming ↑ inflammation
		HDAC2	-	Loss linked to steroid resistance
		IL10	*Acinetobacter lwoffii* (implicated)	IL-6-dependent epigenetic activation reduces asthma susceptibility
		IFNG promoter (H4 acetylation)	*Acinetobacter lwoffii* (implicated)	Stabilized acetylation via TLR signaling in mouse offspring
		Chromatin (via protein VII)	*Adenoviruses* (implicated)	Alters chromatin structure, enhances proinflammatory state
		ORMDL3	-	Hyperacetylation → airway remodeling
		KDM5B (H3K4 demethylation)	RSV (implicated)	↓ KDM5B → ↑ Th1, ↓ Th2 → improved balance
		HOX genes	RSV (implicated)	NS1 interaction with H2BD → HOX activation → lung remodeling
	T2-low	STAT3	-	Altered histone signaling via STAT3 in neutrophilic inflammation
miRNA	T2-high	miR-21	RSV (implicated)	↓ PTEN, ↑ PI3K/Akt → inflammation, fibrosis, steroid resistance; increased in asthma; promotes inflammation via PTEN and TGF-β pathways
		miR-126	-	Suppresses proinflammatory genes; reduced in asthma
		miR-146a	-	↓ NF-κB (TRAF6, IRAK1) → ↓ inflammation, ↑ Treg phenotype
		miR-155	*Rhinovirus* (implicated)	↑ Th1/Th2 responses → ↑ inflammation, asthma severity

**Table 4:** Epigenetics-based candidate therapeutic approaches against asthma.

Full Name	Role	Therapeutic Target	References
DNA Methyltransferases (DNMTs)	Enzymes responsible for adding methyl groups to DNA, contributing to hypermethylation and gene silencing.	Inhibition by DNMT inhibitors (DMNTi) like 5-azacytidine to reverse silencing of anti-inflammatory genes.	[12,15,24,28,37,38,41,83,131,135,136,141,148,153,155,163,174,175,176,179,198,206]
Histone Deacetylases (HDACs)	Enzymes that remove acetyl groups from histones, leading to tighter chromatin and gene silencing.	Inhibition by HDAC inhibitors like vorinostat (SAHA) to promote gene expression and reduce inflammation.	[38,51,53,63,83,135,137,141,142,143,144,145,146,147,148,149,151,152,153,154,164,201,202]
MicroRNA-21 (miR-21)	miRNA upregulated in asthma, associated with inflammation and exacerbations, particularly after viral infections.	miR-21 inhibitors to reduce inflammation and improve lung function.	[12,13,16,27,28,37,38,49,50,63,64,106,136,141,142,143,148,154,158,160,161,164,206,207]
MicroRNA-146a (miR-146a)	Regulates Toll-like receptor (TLR) signaling pathways and inflammatory cytokine production.	Inhibitors to modulate immune responses, particularly in T2-high asthma.	[64,158,165,166,167,168,172,174,182,183,198]
Probiotic strains (e.g., *Lactobacillus*, *Bifidobacterium*, and *Saccharomyces* species)	Probiotic strains that modulate DNA methylation and histone modifications to balance proinflammatory and anti-inflammatory immune responses.	Normalizing hypermethylation of anti-inflammatory genes and promoting histone acetylation for immune tolerance.	[95,153,189,190,191,192,193,194,195,196,197,198,199,203,204,205]
CRISPR-Cas9	Gene-editing technology that targets DNA methylation sites or miRNA binding regions contributing to asthma.	Editing specific genetic and epigenetic alterations in asthma-related endotypes.	[52,68,147,206]
T2-high asthma (Epigenetic Endotype)	DNA methylation of anti-inflammatory genes; miR-21 and miR-146a dysregulation.	DNMT inhibitors, miRNA inhibitors, and immune-modulating probiotics.	[2,18,20,22,27,37,40,41,43,84,87,109,112,113,114,118,119,120,132,133,134,145,147,148,150,151,153,154,155,156,157,158,172,173,182]
T2-low asthma (Epigenetic Endotype)	HDAC-mediated histone modifications.	HDAC inhibitors to alleviate corticosteroid-resistant inflammation.	[2,11,18,20,27,37,39,40,41,42,43,50,84,87,88,109,118,146,148,153,154,155,158,173,182]
